# Multiparametric autoantibody profiling of patients with systemic sclerosis in Greece

**DOI:** 10.31138/mjr.29.3.120

**Published:** 2018-09-27

**Authors:** Christos Liaskos, Emmanouela Marou, Theodora Simopoulou, Athanasios Gkoutzourelas, Maria Barmakoudi, George Efthymiou, Thomas Scheper, Wolfgang Meyer, Christina G. Katsiari, Dimitrios P. Bogdanos, Lazaros I. Sakkas

**Affiliations:** 1 Department of Rheumatology and Clinical Immunology, Faculty of Medicine, School of Health Sciences, University of Thessaly, Larissa, Greece,; 2 Institute of Immunology affiliated to Euroimmun AG, Lübeck, Germany

**Keywords:** autoantibodies, autoimmune disease, diagnostics, scleroderma

## Abstract

**Background:**

Systemic sclerosis (SSc) is an autoimmune rheumatic disease characterized by a wide range of disease-specific and disease-related autoantibodies (autoAbs). Profile assays have been developed and are currently in use to meet the demand for better characterization of all autoAbs found in SSc patients.

**Aim:**

To assess the clinical relevance of SSc-related autoantibodies in 158 patients with SSc, all from Central Greece, taking advantage of a multiparametric SSc autoantibody line immunoassay.

**Material and methods:**

158 consecutive patients with SSc (137 females, mean age 53.2 ± 10 years; 63 patients with dcSSc and 95 with lcSSc) from central Greece were included in the study. Eighteen patients with morphea were also included. Serum samples were analyzed by a profile SSc nucleoli line assay (Euroimmun) to detect Abs against 13 autoantigens: Scl-70, Centromere (A, B), RNA polymerase III (subunits 11 & 155), fibrillarin, NOR90, Th/To, PM/Scl 100, PM/Scl75, Ku, PDGFR and Ro52. Antinuclear autoAbs (ANAs) were detected by indirect immunofluorescence.

**Results:**

ANAs were detected in 97.5% of SSc patients. Reactivities to specific autoantigens were as follows: Topo I, 40.5%; CENP, 32.9%; Ro52, 21.5%; RP11, 8.9%; RP155, 13.3%; NOR 90, 4.4%; Ku 3.8%; PM-Scl75, 3.2%; PM-Scl100, 1.3%; Th/To, 1.3%; Fibrillarin, 1.3%; PDGFR 0%; Ro52 21.5%. Twenty-one of SSc did not have any of the main autoAbs, namely anti-Topo I, anti-CENP, anti-RNA pol III Abs.

**Conclusions:**

Multiparametric autoAb test provides positive SSc-associated autoAb reactivities in SSc patients negative for the three main autoAbs and this may prove of significance in early disease diagnosis.

## INTRODUCTION

Systemic sclerosis (SSc) is an autoimmune disease characterized by widespread fibrosis and microvasculopathy, the latter being exemplified by Raynaud’s phenomenon and digital ulcers.^1^ Although inflammation is not a cardinal clinical feature of SSc, there is plenty of evidence for T cell activation and B cell activation in this disease^2–5^ with the detection of a plethora of autoantibodies (autoAbs), both disease-specific and disease-related autoAbs.^6–9^ Antinuclear antibodies (ANA) are present in nearly all patients with SSc, but certain antinuclear autoAbs targeting specific nuclear autoantigens are associated with distinct cutaneous involvement subsets, namely diffuse cutaneous SSc (dcSSc) and limited cutaneous SSc (lcSSc), cardinal clinical features of the disease.^5,9–13^ These autoAbs known for years are anti-topoisomerase I (anti-Topo I, formerly known as anti-Sc70) antibodies (Abs), and anticentromere Abs, respectively.^5, 9–13^ Furthermore, these autoAbs which are specific for SSc, are present for several years before the clinical onset of the disease, and are very helpful in early diagnosis of SSc.^8,12^ Other disease-specific autoAbs are also associated with other disease manifestations.^14,15^ This prompted immunodiagnostic companies to develop multiparametric assays in the form of dot or line immunoassays which can provide an autoAb profile with a wide range of autoAbs.^6,14^ AutoAbs detected with these assays - including, apart from the conventional anticentromere Abs (anti-CEN) - against centromere protein B (CENPB) and CENPA, and anti-topoisomerase I (anti-Topo I), other less frequent disease-specific autoAbs, such as those against RNA polymerase III (RNA pol III) RNP11 and RNP155 subunits, anti-fibrillarin Abs (also known as anti-U3 ribonucleoprotein abs), anti-PM-Scl (polymyositis-scleroderma) Abs, against 100 and 75 kDa subunits (PM/Scl100 and PM/Scl75), anti-Th/To Abs, anti-nucleolus-organizing region (NOR) 90 Abs, anti-Ku Abs, anti-platelet-derived growth factor receptor (PDGFR) and anti-Ro52 Abs.^14,15^

Recently, it has become apparent that profile assays, which permit testing of several SSc-related Abs, can provide valuable information regarding the diagnostic and clinical relevance of autoAbs in SSc. While anti-CEN, anti-Scl70 and anti-RNA pol III remain by far the most important autoAbs for the diagnosis of the disease,^6,14^ the presence of other autoAbs has been associated at a variable degree with clinical features of the disease such as pulmonary fibrosis, pulmonary hypertension and renal crisis, raising the expectation that they can be used as prognostic markers rather than epiphenomena of immunedysregulation.^12,16,17^

AutoAb testing of SSc cohorts in Greece had been so far limited to few autoantigens (mainly CENP and Scl-70), which are routinely detected by most laboratories, and information regarding reactivity to other autoantigens remained largely unknown. Recently, we took advantage of a commercially available SSc autoAb profile assay and tested a cohort of SSc from our centre for reactivity against 13 SSc-related autoantigens.^6^

Our cohort included homogeneous Caucasian patients^6^ without racial, ethnic or geographical differences, which appear to influence autoAb variation around the globe.^18–23^ Our Department oversees patients not only from the region of Thessaly but also from the surrounding area (Central Greece). We decided to undertake a larger study: we increased the number of SSc patients tested and importantly we included a distinct cohort of patients with morphea, a localized form of scleroderma without involvement of internal organs, a cohort not previously assessed.

## MATERIAL AND METHODS

### Patients

One hundred and fifty-eight patients with SSc (137 females, mean age 53.2 ± 10 years; mean duration of disease 10.6 ± 7.1; 63 patients with dcSSc; 95 patients with lcSSc). Eighteen patients with local morphea (14 female, mean age 42.4 ± 12.7 years) were included as pathological controls. The main clinical characteristics of the patients (morphea and SSc) are shown in **Tables 1** and **Table 2**. Conventional immunosuppressive regimens of SSc patients included low-dose steroids (≤ 7.5 mg/day) plus azathioprine or methotrexate. All patients attended the Outpatient SSc Clinic of the Department of Rheumatology and Clinical Immunology, at the University General Hospital of Larissa, Thessaly, in central Greece,^6,16,17^ and fulfilled the 2013 American College of Rheumatology criteria for SSc.^24^ A written informed consent was obtained by all participants in the study. The study protocol was approved by the Local Ethical Committee of the University General Hospital of Larissa, Greece.

**Table 1:** Major clinical characteristics of 18 patients with morphea included in the study

Age (median, range), yrs	(48.8, 31–75)

Gender (female/male)	14/4

Disease duration (mean ± SD) ( yrs)	3.8 ± 1.1

Morphea subtype:	
Linear	0 (0%)
plaque	15 (82.3%)
generalized plaque	3 (16.7%)

Treatment:	
Topical	14 (77.8%)
systemic	2 (11.1%)
none	2 (11.1%)

**Table 2:** Main clinical features of 158 patients with SSc included in the study

	**Total SSc patients n=158**
**SSctype**	
** lcSSc (n,%)**	95 (60.1)
** dcSSc (n,%)**	63 (39.9)
**Pulmonary Fibrosis (n,%)**	48 (30.4)
**Pulmonary Arterial Hypertension (n,%)**	23 (14.6)
**Ulcers (n,%)**	62 (39.2)
**GI involvement**	
** Upper (n,%)**	91 (57.6)
** Lower (n,%)**	2(1.3)
** Both (n,%)**	8 (5)
**Arthritis (n,%)**	34 (21.5)
**Serositis (n,%)**	10 (6.3)
**Telangiectasia (n,%)**	84 (53.2)
**Calcinosis (n,%)**	18 (11.4)
**RenalCrisis (n,%**)	2 (1.3)

lcSSc: limited SSc, dcSSc: diffused SSc, GI: Gastro-Intestinal

### Methods

Serum aliquots kept at −80°C were used for autoAb testing. AutoAb testing was performed using a line immunoassay (IgG Systemic sclerosis [Nucleoli], Euroimmun Euroline profile kit, Lübeck, Germany) which tests autoAbs against 13 autoantigens.^6^ Topo I, CENPA, CENPB, RP11, RP155, fibrillarin, NOR90, Th/To, PM-Scl100, PM-Scl75, Ku, PDGFR and Ro52. Native Topo I is purified from bovine and rabbit thymus; CENPA, CENPB, PM-Scl100, PM-Scl75, and Ro52 are expressed in insect cells; PDGFR is expressed in mammalian cells; RP11, RP155, fibrillarin, NOR90, and Th/To are expressed in *E. coli*.^6^ All serum samples were tested at a dilution of 1:100 and procedures were previously described in detail.^6^ AutoAb levels were expressed in arbitrary units (AU), and the cut off value for autoAb positivity was set at 11 AU, as previously described.^6^ The major Clinical associations of all SSc-Abs mentioned above, according to the literature are shown in supplementary table

### Statistical analysis

Variation of autoAb levels in each group was defined by standard deviation (SD). Differences between SSc patients and pathological or controls were tested by two-tailed *t*-test, one-way analysis of variance (ANOVA) and the nonparametric Mann-Whitney test. *P*-values smaller than or equal to 0.05 were considered significant. The statistical calculations were performed with Graph Pad Prism Software 5.

## RESULTS

### AutoAb profile in SSc

**Figure 1.**
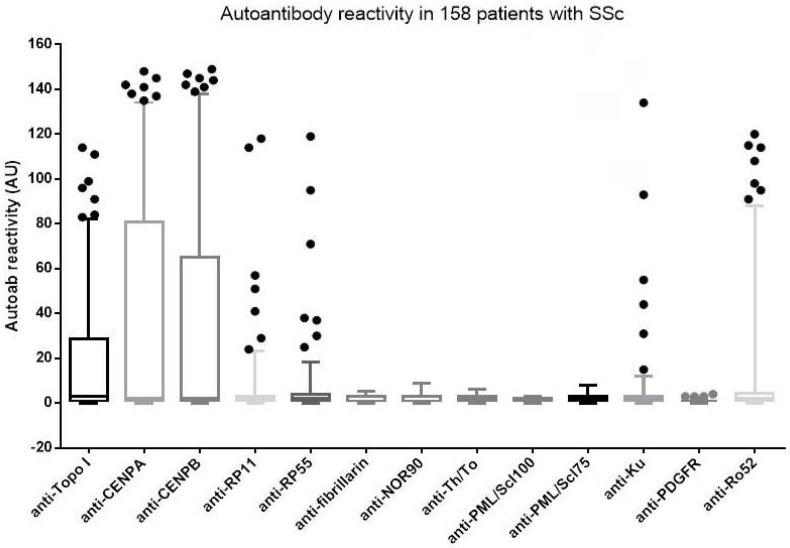
AutoAb levels (expressed as AU) in 158 patients with systemic sclerosis. The solid black line at the center of each box is the median. The arms of each line extend with their ends corresponding to 10 % and 90% of the values.

**Figure 2.**
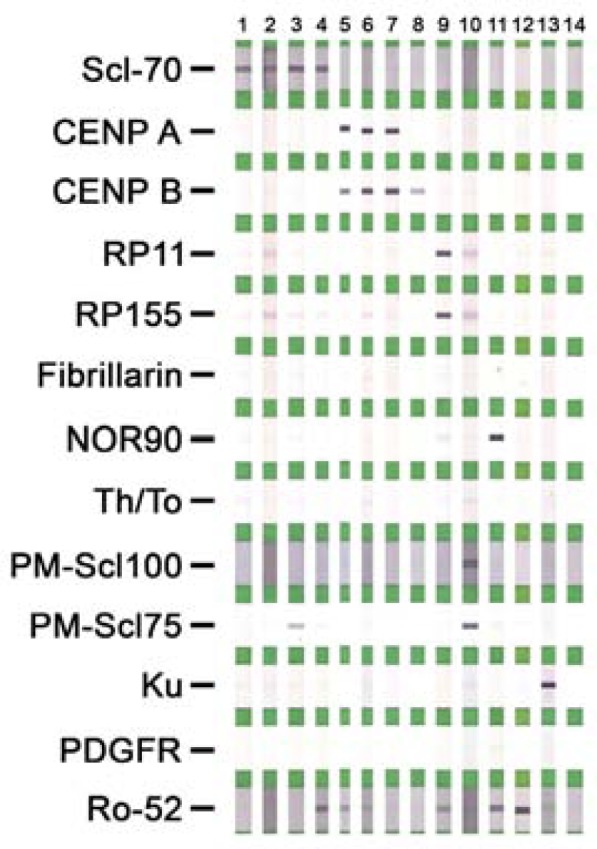
Representative autoantibody patterns of SSc-related autoantibody line assay

Nearly all (97.5%) patients with SSc were positive for ANA by indirect immunofluorescence (IF) on HEp-2 cells (Euroimmun), and the vast majority (95.6%) had autoAbs against at least one autoantigen. AutoAb levels to specific autoantigen in patients with SSc are shown in **Figure 1**, and specific AutoAb reactivity in representative cases is shown in **Figure 2**. Anti-Ro52 Abs, not disease specific, were present in 21.5% of patients. AutoAbs to at least one of the 12 SSc-associated autoantigens (excluding anti-Ro52) were found in 88.6 % of patients. Amongst 18 patients without autoAbs against any specific autoantigen, 14 (77.8%) had ANAs by indirect IF and4 patients (2.5%) lacked any autoAb.

**Figure 3.**
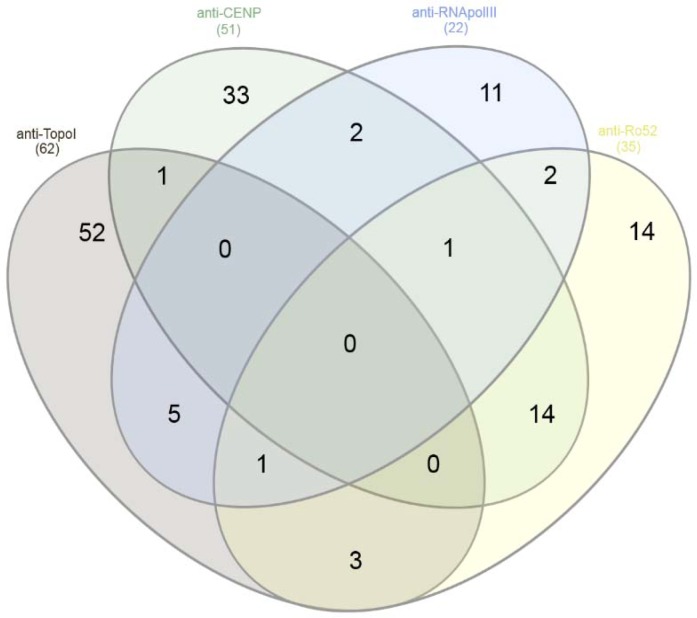
A Venn diagram depicting autoAb positivity of the four most frequent reactivities (anti-Topo I, anti-CEN, anti-RNA pol III, and anti-Ro52)

The frequency of autoAbs in SSc is shown in **Table 3**. Anti-Topo I abs were present in 64 (40.5%) SSc patients (lcSSc, 26.3%; dcSSc, 61.9%) anti-CENP were present in 52 (32.9%), all anti-CENPA and anti-CENPB-positive, SSc patients (lcSSc, 46.3%; dcSSc, 14.2%), anti-Ro52 in 21.5%, anti-RP155 in 21 (13.3%), anti-RP11 in 14 (8.9%), anti-Ku in 6 (4.6%), anti-NOR90 in 7 (4.4%), anti-PM-Scl75 in 5 (3.2%), anti-PM-Scl100 in 2 (1.3%), and anti-fibrillarin in 2 (1.3%), and anti-Th/To in 2 (1.3%) SSc patients. Anti-PDGFR abs were not detected in any SSc patient. Overall, autoAbs to at least one of the three main SSc-associated anti-TopoI (Scl-70), anti-CENP (CENPA or CENPB) or anti-RNApol III (RP11 and/or RP155) autoAbs were present in 125 (79.1%) SSc patients. **Figure 3** shows in the form of a Venn diagram the presence of the four most frequent autoAbs (anti-Topo I, anti-CEN, anti-RNA pol III and anti-Ro52 abs). Fifteen (9.5%) SSc patients, who lacked those three autoAbs, were positive for at least one of the other 9 autoAbs (not including anti-Ro52 reactivity). In 11 patients (6.5%), the only autoAb to specific autoantigen was anti-Ro52. Amongst the 18 patients with morphea, 1 (5.6%) had detectable anti-CENP Abs and 2 (11.1%) had anti-Ro52 Abs.

**Table 3.** Frequency of antigen-specific antibody responses in patients with systemic sclerosis (SSc) total, lcSSc or dcSSc

**AutoAb targets (n, %)**	**Total SSc (n=158)**	**lcSSc (n=95)**		**dcSSc (n=63)**		**P lcSSc*vs* dcSSc**	**P FlcSSc*vs* FdcSSc**	**P MlcSSc vs MdcSSc**	**P FlcSSc*vs* MlcSSc**	**P FdcSSc*vs* MdcSSc**
		**F (87)**	**M (8)**	**F**	**M**					
				**5013**						
**Scl70**	64 (40.5)	22 (25.3)	3 (37.5)	29 (58)	9 (76.9)	**<0.001**	**<0.001**	0.164	0.430	0.337
**CENP**	532 (32.9)	40(46)	4(50)	9 (18)	0 (0)	**<0.001**	**<0.001**	**0.012**	1.000	0.184
**RP11**	14 (8.9)	7 (8)	2 (25)	2 (4)	3 (25)	1.000	0.486	1.000	0.166	**0.055**
**RP155**	21 (13.3)	11(12.6)	1 (12.5)	6 (12)	3 (23)	0.813	1.000	1.000	1.00	0.376
**Fib**	2 (1.3)	2 (2.3)	0(0)	0 (0)	0 (0)	0.517	0.533	na	1.000	na
**NOR90**	7 (4.4)	3(3.5)	3 (37.5)	0 (0)	1 (7.7)	0.244	0.300	0.253	**0.007**	0.206
**Th/To**	2 (1.3)	0 (0)	0 (0)	1 (2)	1 (7.7)	0.157	0.365	1.000	na	0.373
**PM-Scl100**	2 (1.3)	2 (2.3)	0 (0)	0 (0)	0 (0)	0.517	0.533	na	1.00	na
**PM-Scl75**	5 (3.2)	3(3.5)	0 (0)	2 (4)	0 (0)	1.000	1.000	na	1.00	1.00
**Ku**	6 (3.8)	5(5.7)	1 (12.5)	1 (2)	0 (0)	0.244	0.415	0.381	0.419	1.00
**PDGFR**	0 (0)	0 (0)	0 (0)	0 (0)	0 (0)	Na	na	na	na	na
**Ro52**	34 (21.5)	18(20.7)	1 (12.5)	13 (26)	2 (14.4)	0.693	0.527	1.000	1.000	0.716

na, non-applicable, lcSSc: limited SSc, dcSSc: diffused SSc, FlcSSc: female with limited SSc, FdcSSc: female with diffused SSc, MlcSSc: males with limited SSc, MdcSS: males with diffused SSc.

### Associations of autoAbs with clinical features in SSc

Anti-Topo I Abs were more frequent in male (61.9%) than female (37.2%) SSc patients (p=0.032). They were associated with dcSSc (p<0.001), correlated with interstitial lung disease (ILD) (p<0.001) and tended to be associated with digital ulcers (p=0.051). No association was found between anti-Topo I abs and pulmonary arterial hypertension (PAH) (p=0.091). Anti-Topo I abs were more frequently present in males with anti-RP11 abs (23.8%) than female patients (6.6%; p=0.010). No difference in the frequency of anti-RP55 abs between male and female patients were found. Anti-CENP abs were positively associated with lcSSc (p<0.001) and negatively associated with ILD (p<0.001) and the presence of digital ulcers (p=0.045). Anti-NOR90 Abs were more frequent in male SSc patients (male 19% vs female 2.2%, p=0.006) and correlated with ILD (5/7 ILD cases, p=0.016). Anti-Ro52 Abs were associated with arthritis (54.5% vs 17.1%, p=0.027).

## DISCUSSION

Our study reports on the autoAb profile with 13 autoAb reactivities in a well-defined cohort of SSc patients followed up in a reference center in Central Greece. This study, which is an extension of our previous report,^6^ included more patients and assessed a cohort of patients with morphea, a group not previously included in our assessment.^6^

Our results clearly demonstrate the well-recognized dominance of anti-Topo I (Scl-70) autoAbs (62%in dcSSc), and that of anti-CEN (46% in lcSSc) autoAbs in SSc patients,^9,11^ including those from Greece.^6^ Anti-Ro52 Abs remain the third most common autoAb in patients with SSc, being present in more than a fifth of patients affected with this disease.^6,25^ All other autoantibodies, excluding anti-RNA pol III abs which are present in approx. 13% of the patients, are infrequently found. Most profound was the finding that anti-PDGFR Abs were undetectable, despite the fact that we tested one of the largest single center cohorts so far assessed.^26–28^ Whether such a lack of reactivity is due to the decreased sensitivity of the assay (cut-off used for positivity) or is influenced by geographic/ethnic factors remains to be seen.^18–23,29,30^ The fact that the PDGFR used in the current assay is eukaryotically expressed in mammalian cells argues against the possibility that the lack of reactivity is due to the low antigenicity of the antigen source.

Of diagnostic relevance, and in agreement with our previous reported findings, more than 20% of the SSc patients had positive autoAb tests other than Topo I, anti-CEN, or anti-RNA pol III, clearly demonstrating the need to incorporate such profile testing in those patients found negative for the conventional SSc autoAbs.^6,14^ This becomes more important, if we consider that anti-RNA pol III Abs appear to be the only Ab reactivity present on several occasions. Of interest, many laboratories do not test for anti-RNA pol III (mainly RP11 and RP155) Abs and limit their testing just to anti-Topo I and anti-CEN Abs. The take-home message of our findings could be to incorporate SSc autoAb profile testing as a routine test in SSc. Alternatively, one may initially order for anti-CEN, anti-Topo I and anti-RNA pol III Abs and, if tests are negative but the clinical suspicion remains high, to assess for the remaining autoAb specificities.^6,14^ Of interest, autoAb profiling of morphea patients clearly demonstrates that the autoAb pattern in this entity is limited to the presence of anti-CEN and anti-Ro52 in very few patients.

Our study provided a comprehensive analysis of 13 SSc related abs in patients with morphea. Only 2 of the patients had detectable SSc-related abs; 1 double positive for anti-CEN and anti-Ro52, and 1 anti-Ro52 positive. Our data are comparable to previous study showing an infrequent/rare detection of SSc related abs in patients with morphea.^31^ Nevertheless, meticulous assessment of such abs needs to be performed in a large, most likely multicenter, study. In respect to anti-Ro52 Abs, an increasing number of recent studies reports a high frequency of these autoAbs in SSc, but their clinical relevance remains uncertain, as most studies fail to identify clinical usefulness attained by this autoAb.^25,32,33^ Whether their appearance is an epiphenomenon or pathogenically relevant remains a matter of debate and the topic of intense investigation.^34^

AutoAb reactivities noted in patients with SSc have been associated with specific clinical phenotypes.^5,8–12^ The prevailing dogma, also supported by our findings, is that anti-Topo I Ab seropositivity is largely associated with dcSSc and that of anti-CEN with lcSSc.^5,6,8–12^ In addition to that, several other clinical associations were noted and need to be commented on. We confirmed the previously noted association of anti-Topo I Ab positivity with ILD (p<0.001), and at the other end, the negative association of anti-CENP Abs with ILD.^8,35^ The fact that anti-Topo I Abs are twice as high in male than in female SSc patients (a feature also seen for anti-RNA pol III and anti-NOR90 abs) is of interest and necessitates further investigation.^22^ The anti-NOR90 Abs, despite its low frequency, was also associated with ILD, and this should not be ignored. Finally, anti-Ro52 Abs were associated with the presence of arthritis, but not with ILD. The tri-nation (Canada, Australia, USA) cohort^25^ which tested the clinical significance of anti-Ro52 Abs in 1,574 SSc patients found that monospecific anti-Ro52 Abs were associated with ILD and poor prognosis.^25^ As we have previously reported, 9.1% of the 22 individuals with Raynaud’s phenomenon and capillaroscopy features inconsistent with SSc, and 13.6% of the NCs respectively, had anti-Ro52 antibodies, and no other autoAb reactivity.^6^ Anti-RNA pol III abs have been associated with scleroderma renal crisis (SRC), but our study included only 2 patients with SRC, where 1 of them had anti-RNA pol III abs.

Finally, one of the autoAbs that most likely need to be tested (but is not included in the commercial strip used) is that of anti-U1-RNP, as this is an important antibody for stratification of these patients, for those with PAH in particular.^36^

## CONCLUSION

In conclusion, our study points towards a better understanding of the autoAb presence in patients with SSc. Multiple parametric testing of several autoAb specificities appears to be an important tool, not only to assess the diagnostic relevance of these autoAbs but also their clinical significance, which remains a topic of debate. A multicenter national study in SSc patients from all over Greece may provide extremely useful information.

## FINANCIAL SUPPORT

ELKE (Special Account for Research Grants), University of Thessaly

## Figures and Tables

**Supplementary Table 1:** Major clinical associations of SSc-related autoantibodies.^26, 37–39^

**SSc specific autoantibodies**	**Antigen target**	**Clinical Associations**
Scl-70	DNA topoisomerase I	Diffuse form of SSc, Digital Ulcers, ILD
CENPA and CENBP	Centromere protein A and B	Limited form of SSc, lower risk for SRC
RN11 and RN 155	RNA polymerase III, subunits 11 and 155	Renal Crisis, malignancy, GAVE, Reduced survival
U1-RNP	U1 small nuclear ribonucleoprotein 70 kDa	PAH, MCTD
Fibrillarin	U3-RNP	Diffuse form of SSc, PAH, overlap with myositis or SLE
NOR90	a 90-kDa protein that recognize a component of the nucleolus-organizing region of chromatin	Limited SSc, ILD, Overlap with Sjogren
PM-Scl	Antigen complex of 11–16 polypeptides. Major antigens PM-Scl 75 and PM-Scl100	Digital Ulcers, ILD, Overlap with myositis,
Th/To	7-2RNP/7-2-RNA protein complex	PAH, ILD, limited SSc
Ku	(p70/p80) antigen- a DNA binding protein involved in doubled-stranded DNA repair.	Limited SSc, ILD, increased CK levels
PDGFR	Platelet Derived Growth Factor Receptor	NA

PAH: Pulmonary Arterial Hypertension, ILD: Interstitial Lung Disease, SLE: Systemic Lupus Erythematosus, GAVE: Gastric Antrial Vascular Ectacia, MCTD: Mixed connective tissue disease, SRC: Scleroderma Renal Crisis.

## ABBREVIATIONS

Ab: antibody
ANA: Antinuclear Antibody
AutoAb: autoantibody
SSc: Systemic Sclerosis
